# Observer bias in medical evaluations: a critical pre-analytical factor impacting the quality of judicial evidence in forensic-toxicological casework

**DOI:** 10.1007/s00414-026-03893-z

**Published:** 2026-06-29

**Authors:** Uta Kuepper, Benno Hartung

**Affiliations:** Institute of Legal Medicine, University Hospital Essen, Hufelandstr. 55, 45147 Essen, Germany

**Keywords:** Observer bias, Medical evaluation, Blood sampling, Pre-analytical factor, Correlation, Forensic-toxicological assessment

## Abstract

This study investigates observer variability and systematic bias in medical examinations conducted during forensic blood sampling. The analysis focused on 14 experienced contract physicians routinely employed by law enforcement, a cohort expected to provide the highest degree of diagnostic reliability. In total, raw data from 1458 medical examinations were numerically coded and evaluated.

Nine of the 14 physicians demonstrated signs of systematic bias, characterized by over- or underreporting of impairment symptoms compared to the peer group. Furthermore, a complete lack of (or only highly restricted) correlation between the clinical assessment of impairment and the analytical blood alcohol concentration (BAC) was observed in six practitioners. Adherence to standardized test batteries was equally inconsistent; four physicians completely omitted at least one diagnostically critical motoric test (either explicitly stated or implied), while three others significantly exceeded the average omission rates of the peer group.

Despite state-of-the-art laboratory analytics, these findings indicate that - even among experienced physicians – the forensic utility of the medical report is significantly limited in approximately 40% of cases and entirely useless in an additional 10%. Given the high judicial regard for medical assessments over police observations, this lack of standardization undermines the principle of equal treatment. Furthermore, it hinders research into substance-related safety thresholds. Following the successful optimization of laboratory standards and police training, it is imperative to standardize the medical assessment process through specialized qualifications und proficiency testing. Special consideration should be given to using police bodycam recordings for objective forensic reviews, at least if a criminal offense is suspected.

## Introduction

Police casework involving suspected substance use is inherently complex, spanning from driving under the influence (with or without signs of impaired driving ability) to criminal offenses in which the offender’s diminished capacity may be legally relevant. This investigative process is a multi-stage procedure beginning with the identification of outwardly visible signs of impairment by police officers. During initial contact, officers evaluate indicators such as gait, speech, cognitive processes, and mood. These observations may be supplemented by a battery of psychophysical tests – standardized field sobriety tests – including “beeline”/walk-and-turn, one-legged stand, finger-to-finger test, finger-to-nose test, and Romberg test [1–3], alongside preliminary breath, urine, or oral fluid screenings [4, 5]. If suspicion is substantiated, a blood sample is taken for evidentiary purposes. In Germany, the federal structure of the police system has prevented the adoption of a truly uniform procedure for this process: while recent years have seen increasing efforts to standardize police drug-detection procedures through state- and nation-wide training programs, procedural differences persist – even within federal states.

Although blood sampling generally requires a judicial warrant, a 2017 amendment to German law grants police the authority to order samples in traffic-related cases. This measure aims to prevent the loss of evidence due to the metabolic degradation of substances [6]. In Germany, the clinical part of the procedure is conducted by a physician, who performs a medical evaluation concurrent with the blood draw. Once again, visible signs of substance use (gait, speech/pronunciation, pupils, cognitive function, mood) are assessed, and voluntary tests are conducted to further evaluate the subject’s motor functions and cognitive abilities (turnaround, finger-to-finger test, finger-to-nose test, pupillary response to light). Based on the individual findings, the physician issues a final evaluation of the overall degree of impairment. These findings are documented on a standardized form developed and provided by the federal police, the content of which therefore varies across Germany. In North Rhine-Westphalia (NRW), the medical examinations are preferentially performed by contract physicians; while less experienced medical staff may be utilized based on availability, such instances are rare and do not allow for the acquisition of relevant forensic experience. Furthermore, apart from the form (which serves only as a rough, non-mandatory guide), and the general principles of good medical practice, there is no official guideline or standard operating procedure (SOP) governing the performance and documentation of forensic blood collection.

Following the clinical assessment, the blood sample undergoes analytical toxicology in accredited laboratories, which are required to adhere to DIN EN ISO/IEC 17025 standards to ensure high data quality [7–10]. The resulting analytical and clinical evidence dictates the legal trajectory of the case, including criminal prosecution or administrative sanctions.

In contrast to the systematic efforts of recent years to optimize police roadside testing and the increasingly stringent quality control in laboratories, medical assessments have remained largely unchallenged: Traditionally, German judicial systems place great, often unquestioned trust in these medical findings and, due to the presumed superior diagnostic expertise of physicians, afford them far greater weight than police observations. Given that these evaluation procedures are not grounded in any nationally or internationally recognized standards (see above), it is questionable whether such trust is justified.

This study therefore seeks to retrospectively evaluate the diagnostic validity of medical examinations and to identify any potential systematic biases in the assessments made by panel physicians. To this end, the present work examines whether and to what extent a correlation can be established between clinical assessments and final laboratory results.

As the analytical parameter of choice, the investigation will focus on blood alcohol concentration (BAC) since ethanol exhibits the most predictable dose-response relationship [11] and linear elimination kinetics [12, 13]. To the best of our knowledge, this is the first study to systematically test observer variability in physicians working in a forensic context. It is hypothesized that a strong correlation exists between BAC and medical assessments within this experienced cohort and that no significant systematic examination bias will be observed.

## Materials and methods

### Study population and inclusion criteria

During the 2.5-year observation period from August 1, 2023, to January 31, 2026, the Institute of Forensic Medicine in Essen (Germany) recorded all requests for blood alcohol concentration (BAC) analysis from police departments within its catchment area. The study included cases involving both traffic and other offenses/crimes, provided that alcohol was the sole substance of concern; cases with suspected polydrug use or other exogenous substances were excluded. While most requests involved a single blood sample, cases involving double sampling – typically conducted 30 min apart to address “hip-flask” defenses – were included, though only the BAC from the initial sample was utilized for this analysis. In accordance with German forensic standards, the BAC (expressed as g/kg in whole blood) was defined as the mean of four individual measurements obtained via two distinct analytical methods (usually a combination of an enzymatic method and gas chromatography with a flame ionization detector, i.e. GC-FID), each performed in duplicate. The laboratory is accredited under DIN EN ISO/IEC 17025 and adheres to DAkkS (German Accreditation Body) and GTFCh (German Society of Toxicological and Forensic Chemistry) standards, ensuring that all methods are validated and subject to regular proficiency testing.

### Contents of the “medical report” form

Police procedures in Germany vary by federal state, and this study focuses exclusively on the jurisdiction of North Rhine-Westphalia (NRW), where the medical evaluation is documented on a standardized form developed and provided by the state’s central police agency (Landesamt für Zentrale Polizeiliche Dienste, LZPD). The core of the document is the medical examination section, which utilizes checkboxes for 12 clinical parameters (rotatory nystagmus was excluded from this study due to highly inconsistent reporting), each graded across 2–6 response options. The first option for each category corresponds to the standard findings in unimpaired persons, while the other choices – listed in the same order as on the form – describe the range of potential abnormalities:

- gait: steady, unsteady, staggering, shuffling
- turnaround (after walking): confident, unsteady
- finger-finger-test: confident, uncertain
- finger-nose-test: confident, uncertain
- speech/pronunciation: clear, slurred, garbled
- pupils: unremarkable, (markedly) dilated, (markedly) constricted
- pupillary response: prompt, sluggish/poor, fixed/non-reactive
- consciousness/vigilance: clear, drowsy/somnolent, unconscious, confused (multiple entries allowed)
- impaired orientation or memory (regarding the incident): (no), yes
- thought process: organized, erratic/tangential, persevering, disorganized/incoherent, other (multiple entries allowed)
- behavior: calm/restrained, talkative, disinhibited, rejecting, aggressive, slowed, lethargic, other (multiple entries allowed)
- mood: unremarkable, depressed, euphoric, dull, irritable, other (multiple entries allowed)

Based on the findings in the 12 categories listed above, the physician provides a final assessment of the overall degree of outwardly visible substance influence, categorized as: (none), mild, marked, severe, or gross (intended to represent a gradual increase in impairment severity).

The medical reports are signed and stamped by the examining physician, allowing for individual attribution. This mode of attribution was of crucial importance for this work, as most cases of drunk driving in Germany are decided according to the “per se” principle solely on the basis of test results, and the authors therefore often did not have access to detailed or supplementary case files.

### Data coding and preparation

The global medical assessment of impairment was numerically coded from “1” (mild) to “4” (gross). Ambiguous ratings (e.g., “marked to severe”) were coded as half-steps (e.g., “2.5”). Missing final evaluations were generally marked as “n.a.” (not applicable) and excluded from the correlation analysis. However, if supported by a specific comment indicating the explicit absence of clinical signs (e.g. “none”, “no” etc.), a “0” was assigned.

Additionally, all medical reports from a given practitioner were analyzed for his/her rating profile across the various individual criteria. For this, all positive clinical findings were converted into numerical matrices. A missing entry in a specific category was interpreted as a normal (negative) finding and coded accordingly – the only exception being the impairment of orientation and/or memory, where a lack of dedicated checkboxes for negative findings necessitated manual coding based on qualitative remarks (see above for the global assessment). It is important to acknowledge, however, that this approach does not allow for a definitive distinction between truly negative results and tests that were simply omitted. This limitation stems from inconsistent documentation regarding the frequency and extent of omitted tests across the physician collective (see below).

### Omission rate of motoric tests

The study differentiated between declared and inferred test omission rates. The declared test omission rate (dTOR) was calculated as the percentage of a physician’s total cases in which phrases such as “not performed,” “not possible,” or “rejected” indicated omission of at least one motoric test. Furthermore, an inferred test omission rate (iTOR) of 100% was assumed for a specific parameter if a physician did not assign any ratings for that category across their entire case collective.

### Correlation of BAC and global assessment of alcohol impairment

Correlation analysis was restricted to experienced contract physicians with a minimum caseload of 60 examinations for later BAC analysis during the observation period. Linear regression was selected as the model of choice, as the medical assessment form implicitly suggests a linear relationship between BAC and clinical impairment. To test this expectation, each measured BAC was plotted against the corresponding, medically assessed, and numerically coded degree of alcohol impairment. The resulting strip-plots were supplemented with a trend line drawn through the median BAC values for each impairment level. However, this approach required the physician to have employed at least three different rating levels with a minimum of three data points each, since otherwise it would not have been possible to calculate a meaningful median. Trend lines were explicitly not forced through the origin to account for potential alcohol tolerance, which could cause the intersection with the x-axis to take on positive values. The coefficient of determination (R^2^) was calculated only for physicians utilizing at least four rating levels: this prerequisite was formulated to ensure a baseline level of statistical validity, as regressions based on three or fewer rating points tend to produce artificially inflated R² values of > 0.99.

### Evaluation profile of individual criteria

To assess individual examination styles, the frequency of normal findings for each criterion was determined for each physician within the peer group (caseload > 60). This approach – focusing on the rate of normal findings rather than the frequency of abnormalities – was necessary to prevent statistical distortion, as a single parameter could contain multiple entries for abnormal findings. Practitioners were then screened for systematic under- or overreporting based on their position relative to the group distribution: A percentage of normal findings falling below the 25th (slight bias, + 1) or 10th percentiles (severe bias, + 2) was categorized as overreporting. Conversely, a rate of unremarkable findings exceeding the 75th or 90th percentiles was categorized as slight (-1) or severe (-2) negative bias/underreporting, respectively. Frequencies within the 25th to 75th percentile range were considered unbiased (0).

Furthermore, a cumulative analysis was performed for each physician across all parameters to identify overall trends in over- or underreporting. This was achieved by summing the negative and positive bias scores, respectively. A significant trend toward underreporting was defined as a cumulative negative value of at least − 4, representing either more than two extreme outliers or a consistent negative trend across more than four individual criteria. Analogously, a significant overreporting trend was defined as a cumulative positive value of 4 or higher, indicating either more than two extreme positive outliers or a stable positive trend across more than four individual criteria.

## Results

During the 2.5-year observation period from August 1, 2023, to January 31, 2026, the Institute of Forensic Medicine in Essen recorded a total of 8002 requests from referring police departments. Of those, 2327 cases involved a request for blood alcohol concentration (BAC) analysis without indication of other centrally acting compounds.

### Collective of (contract) assessing physicians

In approximately 5% of all cases, the medical examiner could not be identified from the available documentation. Of the attributable procedures, the vast majority were performed by 39 designated contract physicians, with the remainder conducted by hospital staff or local practitioners. Because the latter handled only isolated cases each, they could not accumulate meaningful forensic expertise. Consequently, these practitioners were excluded from subsequent analysis, as no single individual met the necessary threshold, regardless of the aggregate volume of cases handled by these professional groups. Furthermore, the exclusion of cases managed in hospital emergency rooms (ER) effectively removed any potential influence of severe traumatic injuries from the dataset, as these patients undergo immediate medical intervention rather than routine traffic-related examinations.

**Fig. 1:**
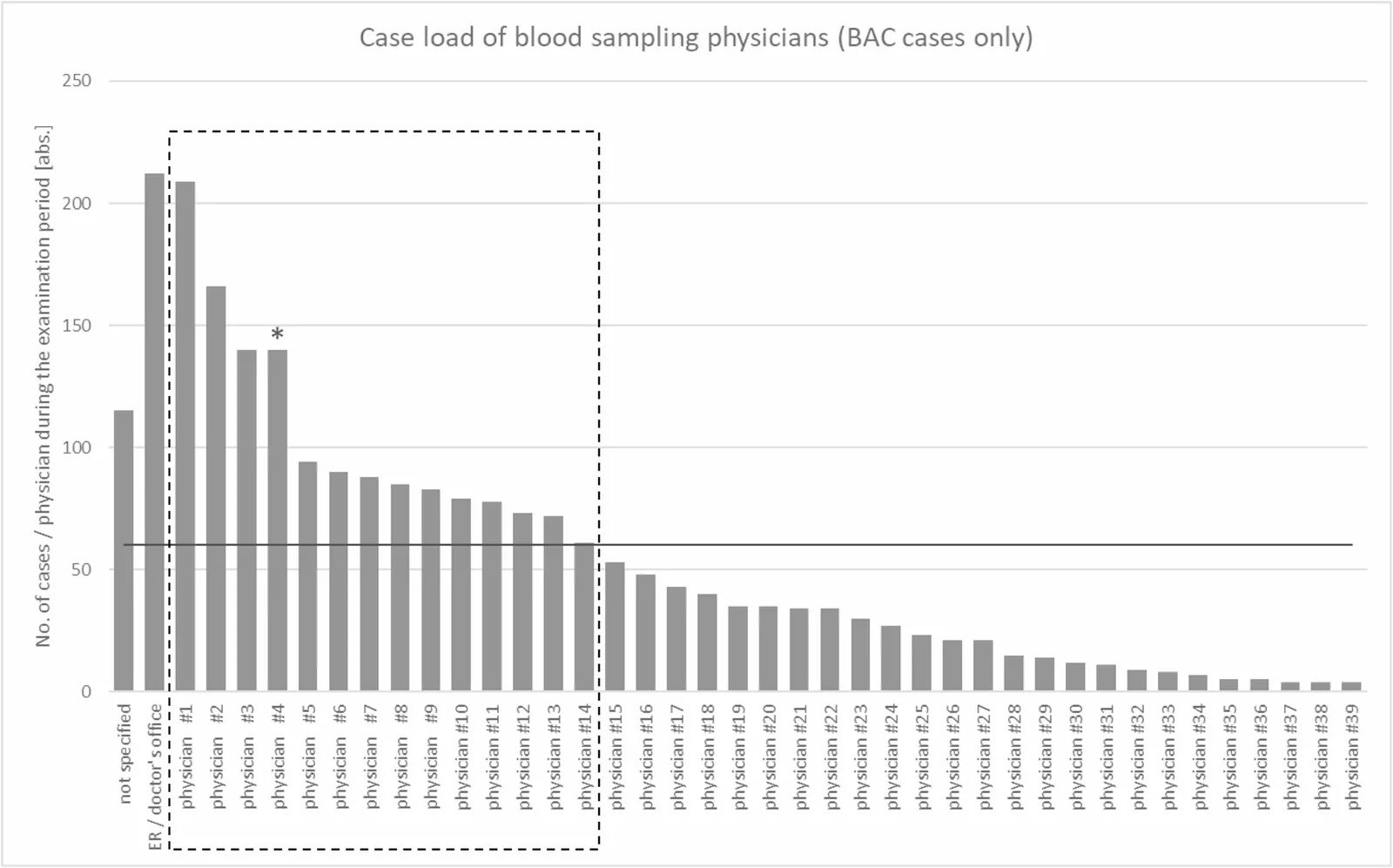
Caseload distribution of medical examiners during the observation period. Data inclusion is restricted to clinical examinations conducted as part of a blood sampling for later BAC analysis. Panel physicians with a caseload of 60 or more examinations (indicated by the *horizontal line*) were categorized as experienced (#1–14, highlighted within the *dotted box*). The *asterisk* (*) identifies a specific practitioner (Physician #4) for detailed discussion in subsequent sections. Not included were datasets from less experienced physicians, i.e. panel physicians with less than 60 examinations, as well as cases from emergency rooms (ER) or local resident practitioners (doctor’s office)

To ensure anonymity, all remaining physicians were assigned numerical pseudonyms in descending order of their total caseload. Contract physicians who conducted 60 or more examinations during the observation period were categorized as experienced; this criterion applied to 14 physicians who collectively performed 1458 procedures (representing 63%, or nearly two-thirds, of all cases). Figure 1 illustrates the caseload distribution across all participating physicians, with the 14 most experienced practitioners highlighted within a dotted box.

### Distribution of blood alcohol concentrations

**Fig. 2:**
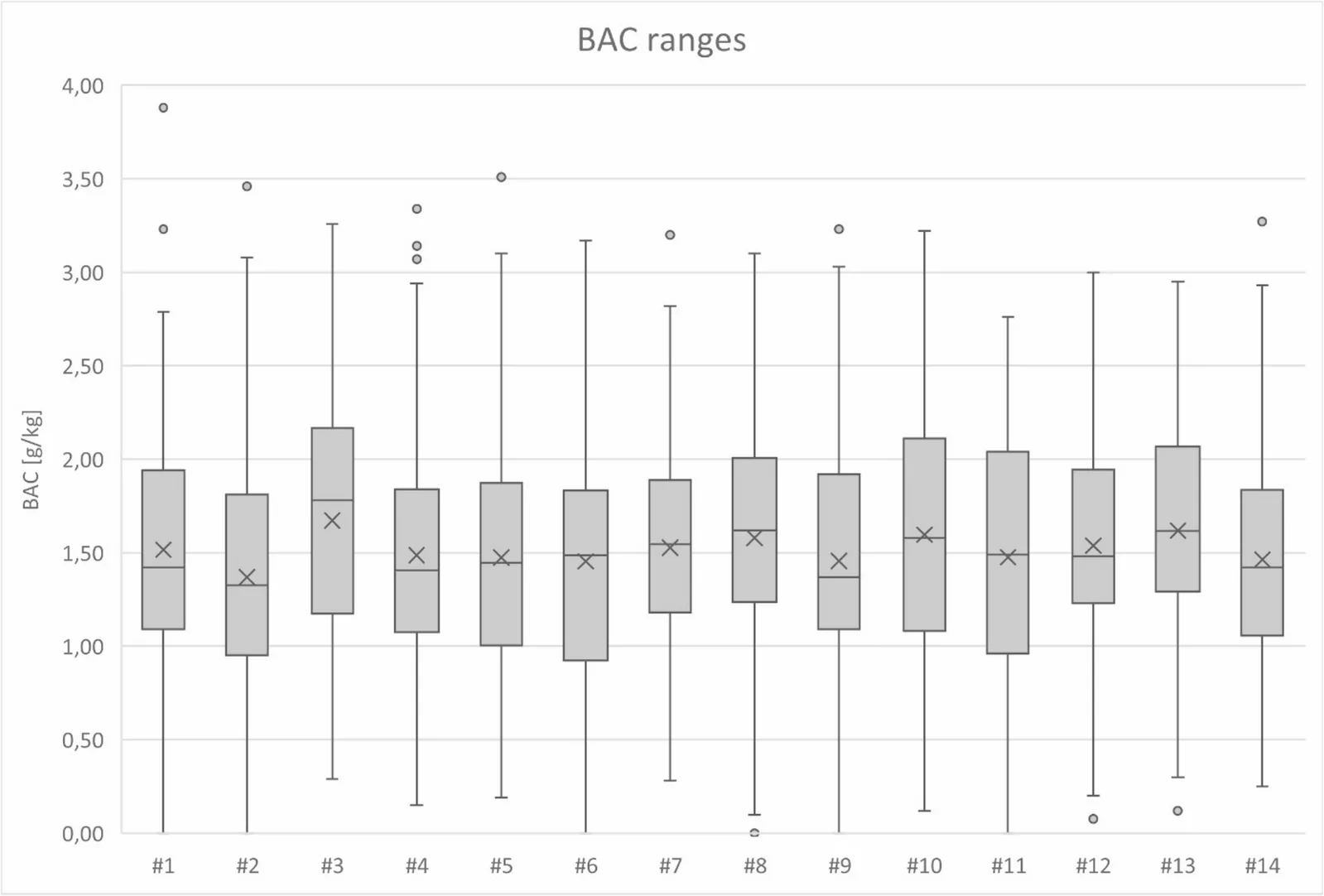
BAC ranges in the subject collectives of the 14 most experienced contract physicians. The boxplots illustrate the distribution of BAC across the subject collectives of physicians with a caseload exceeding 60 assessments. Following the exclusion of physician #3, no statistically significant differences were observed across the subject cohorts of the remaining physicians. *Boxes* contain 50% of the observations. *Black lines* indicate the median. *Crosses* indicate the average values. *Circles* indicate outliers

The variance in blood alcohol concentrations (BAC) across the subject collectives of the individual physicians was statistically evaluated. Median BAC values ranged between 1.33‰ (g/kg) and 1.78‰, with minimum values spanning from 0.00‰ to 0.29‰ and maximum values between 2.76‰ and 3.88‰. Analysis of variance (ANOVA) indicated that these differences across the cohorts were statistically significant (*p* = 0.04; critical F-value: 1.73). Notably, Physician #3 presented a cohort with a substantially higher median BAC of 1.78‰, whereas the medians for all other physicians clustered between 1.33‰ and 1.62‰. Following the exclusion of Physician #3 from the dataset, no statistically significant difference remained in the BAC ranges of the remaining 13 physicians (#1–2 and #4–14; ANOVA, *p* = 0.29; critical F-value: 1.76). Figure 2 illustrates the distribution of blood alcohol concentrations across the subject cohorts for each physician.

### Reporting of individual criteria

**Fig. 3:**
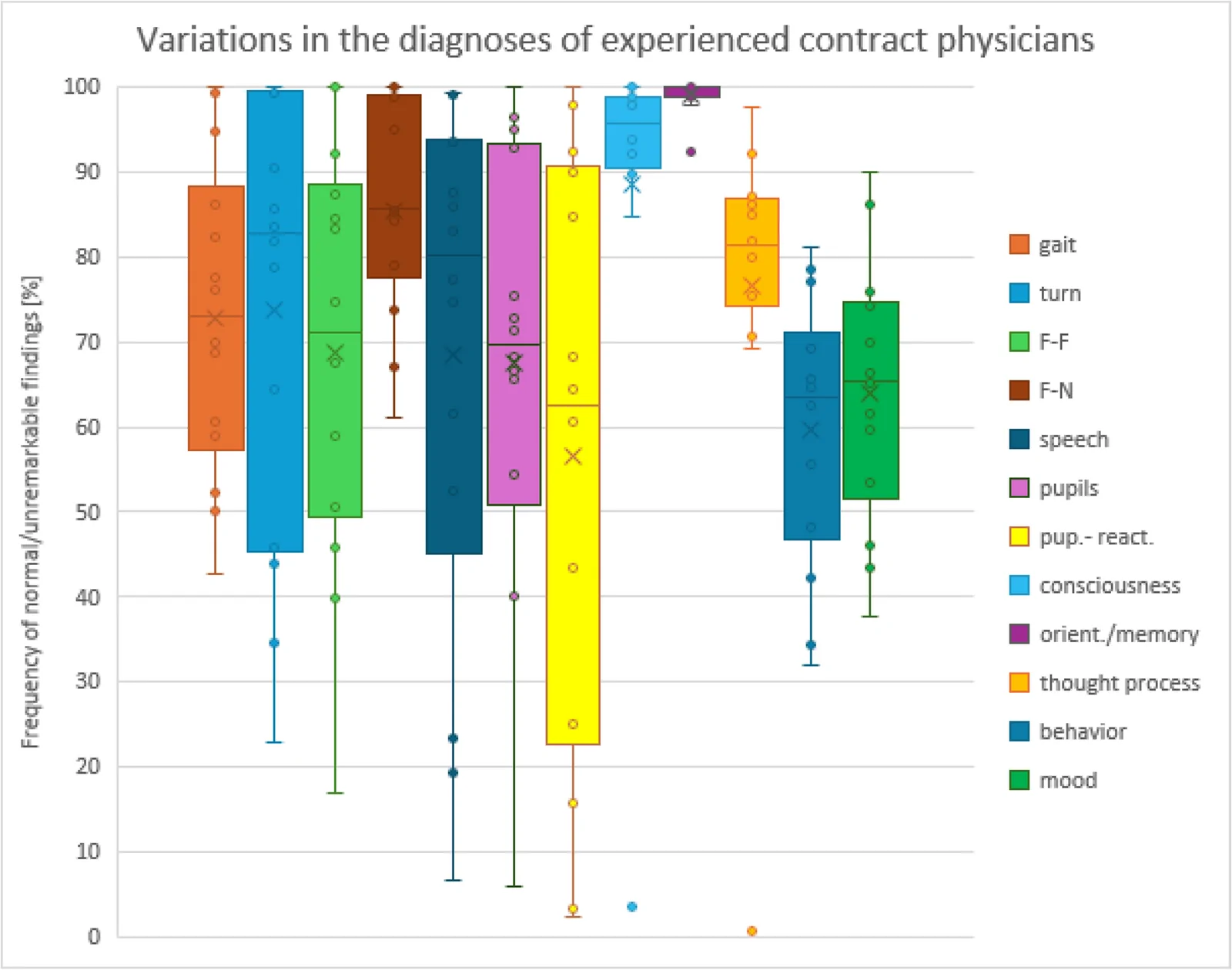
Documentation frequency of normal/unremarkable findings across clinical assessment parameters. *Boxes* represent the interquartile range (middle 50% of the distribution), with *horizontal lines* indicating the median and *crosses* representing the mean values. *Whiskers* extend to the most extreme data points within 1.5 times the interquartile range. Individual data points (*circles*) represent the specific documentation frequencies for each of the 14 physicians. The graph highlights considerable inter-observer variation among the 14 most experienced physicians. Discrepancies are most pronounced in parameters related to motor functions and pupillary characteristics

The documentation of individual clinical criteria exhibited substantial variance across the different examining physicians (Fig. 3). Marked fluctuations in findings were particularly evident in the assessment of motor functions – specifically gait, turnaround, finger-finger test, and speech/pronunciation – as well as in the evaluation of pupillary characteristics.

### Bias in the reporting of individual criteria

The analysis revealed significant deviations in the assessment of individual parameters across the cohort. Six physicians exhibited substantial overreporting – defined by an outlier score of + 2 in at least one clinical parameter (Physicians #2, 4, 5, 8, 9, and 11). Conversely, four physicians demonstrated substantial underreporting (outlier score of -2) in at least one parameter (Physicians #4, 7, 9, and 10).

When examining the cumulative bias of practitioners, five physicians showed no relevant cumulative bias (#1, 10, 12, 13, and 14). However, nine physicians presented with significant cumulative bias: Physician #4 represented the most extreme case, characterized by a unique combination of severe under- and notable overreporting. The remaining eight physicians displayed significant tendencies toward either underreporting (Physicians #3, 5, and 7) or overreporting (Physicians #2, 6, 8, 9, and 11). Table 1 provides a comprehensive overview of these findings, depicting the reporting habits of the individual physicians as a heatmap, where over- and underreporting are indicated by red versus blue hues.

**Table 1:**
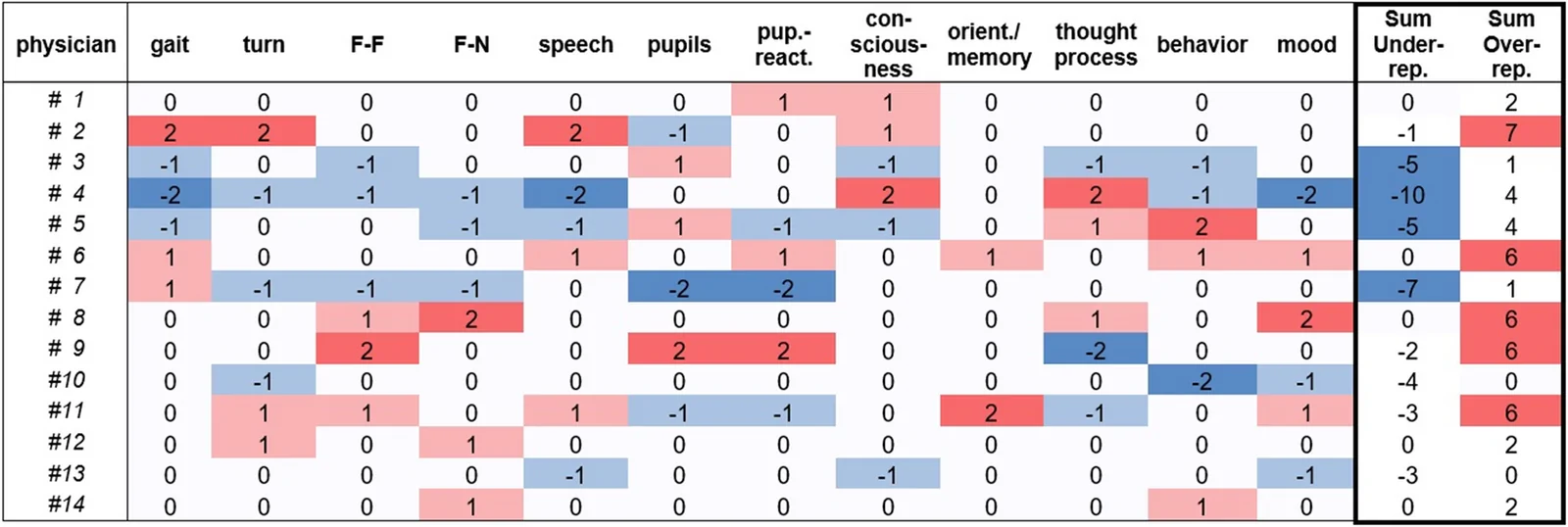
Heatmap of reporting bias across clinical assessment parameters. Negative values indicate slight (–1, light blue) or significant (–2, dark blue) underreporting, whereas positive values (+ 1 or + 2, darkening shades of red) signify slight or significant overreporting, respectively. A value of “0” (white) denotes assessments within the average range of the peer group. The rightmost columns display cumulative sum values for negative and positive bias; scores exceeding ± 4 (indicating more than two outliers or a consistent trend across four or more criteria) are color-coded and considered indicative of relevant directional bias (overreporting in red, underreporting in blue)

**Fig. 4:**
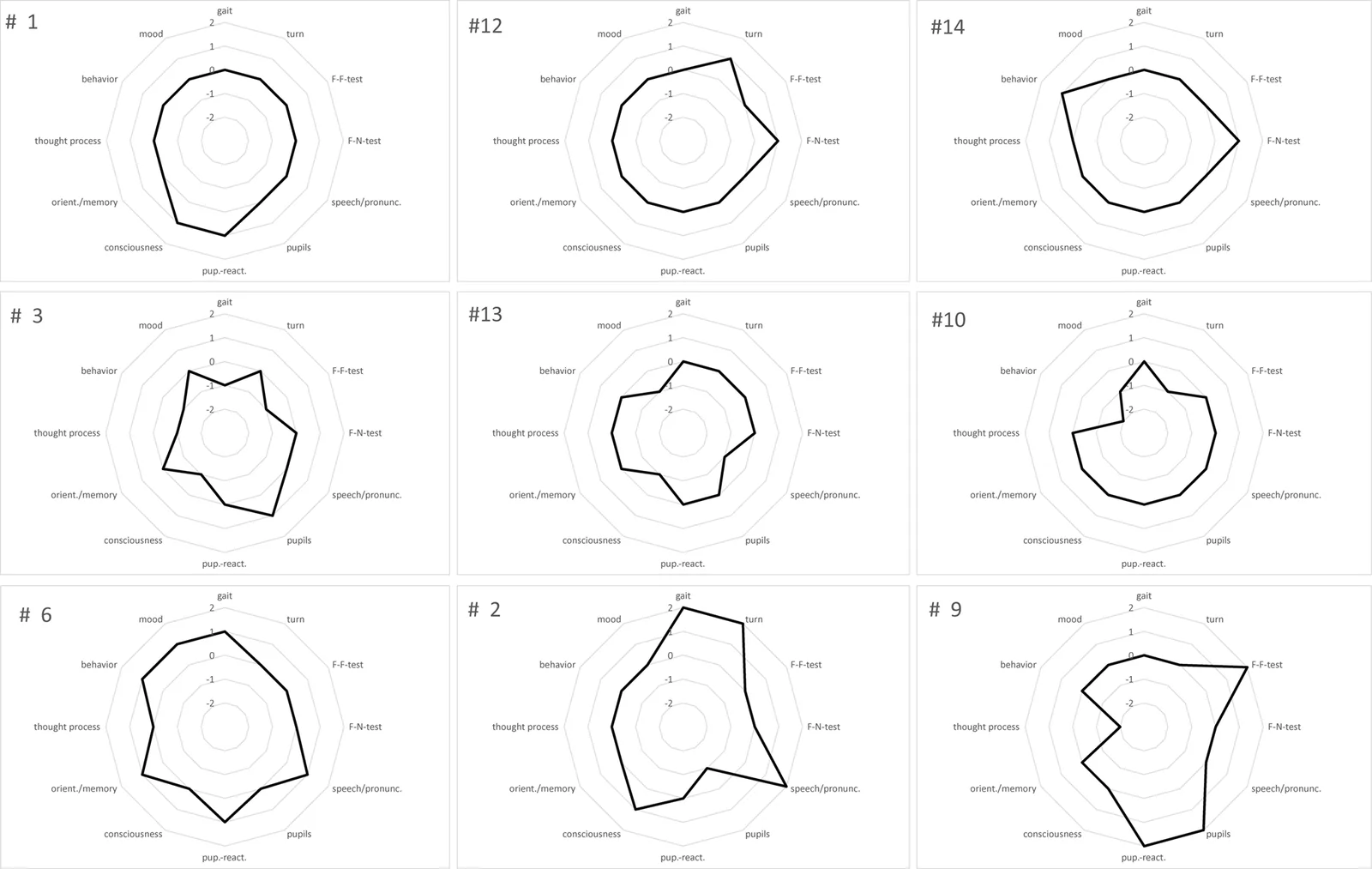
Representative radar charts of individual reporting profiles. A concentric circle at the “0” baseline represents the peer group median (zero bias). The top row displays physicians with the highest concordance to the group average. These are contrasted with profiles exhibiting systematic underreporting (middle row, contracted data polygons) and overreporting (bottom row, expanded polygons)

To further visualize individual reporting styles, radar charts were employed to map physician-specific biases across all assessment parameters (Fig. 4). In this model, an ideal assessment profile – demonstrating full concordance with the peer group average – is represented as a concentric circle at the “0” baseline. Minor deviations (± 1) reflect subtle shifts in an individual’s diagnostic threshold relative to the group average. In contrast, values reaching the innermost or outermost perimeters indicate severe reporting bias. Systematic under- or overreporting across multiple criteria is visually manifested as a contraction or expansion of the total polygonal area, respectively.

### Correlation of BAC and global assessment of alcohol impairment

**Fig. 5:**
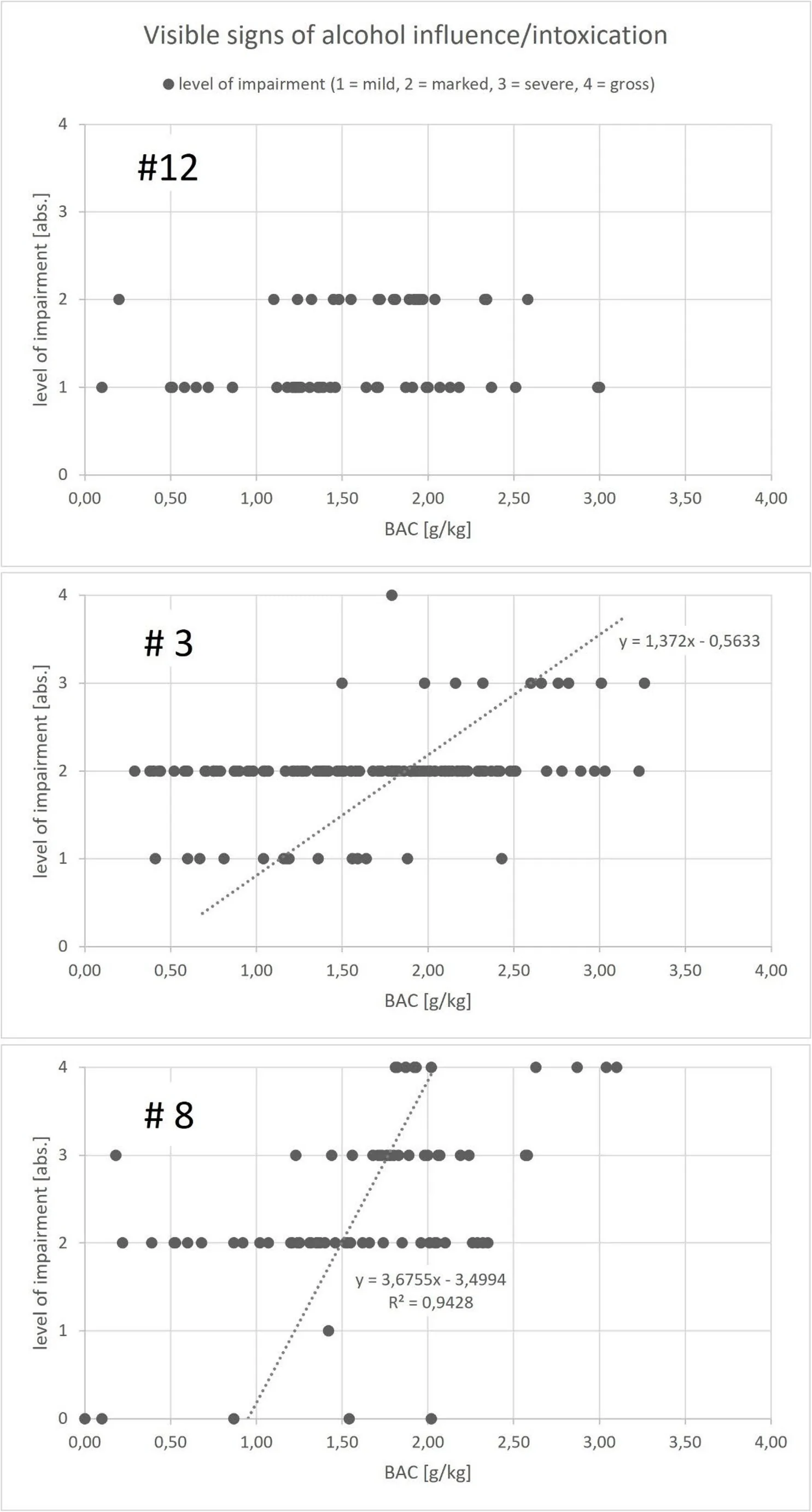
Correlation between global assessment of impairment and forensic BAC across individual physicians. Examples for missing (top) and weak correlations (middle, bottom) due to a biased diagnosis of impairment level are shown. Examples illustrate how systematic reporting bias – such as restricted variance (top) or skewed distribution (middle and bottom) – precludes or weakens the correlation between clinical findings and measured BAC

Significant differences in assessment tendencies were observed among the physicians, characterized by distinct “assessment focal points” (preferred impairment grades) that impacted the feasibility of correlating BAC with clinical evaluations (Fig. 5):

For instance, Physician #12 predominantly utilized only two impairment levels (grades 1 and 2) in a nearly identical distribution, effectively precluding a meaningful correlation. In contrast, Physician #3 focused heavily on grade 2 (within a 1–3 range), with an isolated outlier at grade 4; while a correlation could be established, the data distribution was insufficient for a meaningful calculation of the coefficient of determination (R^2^).

Conversely, physician #8 demonstrated a more balanced distribution across grades 0 and 2–4, allowing for both correlation analysis and determination of R^2^. However, this assessment was notable for a marked underrepresentation of grade 1 and a steep regression slope: the latter suggests a lower threshold for diagnosing higher impairment, leading to a faster transition across escalating impairment levels compared to the peer group.

**Fig. 6:**
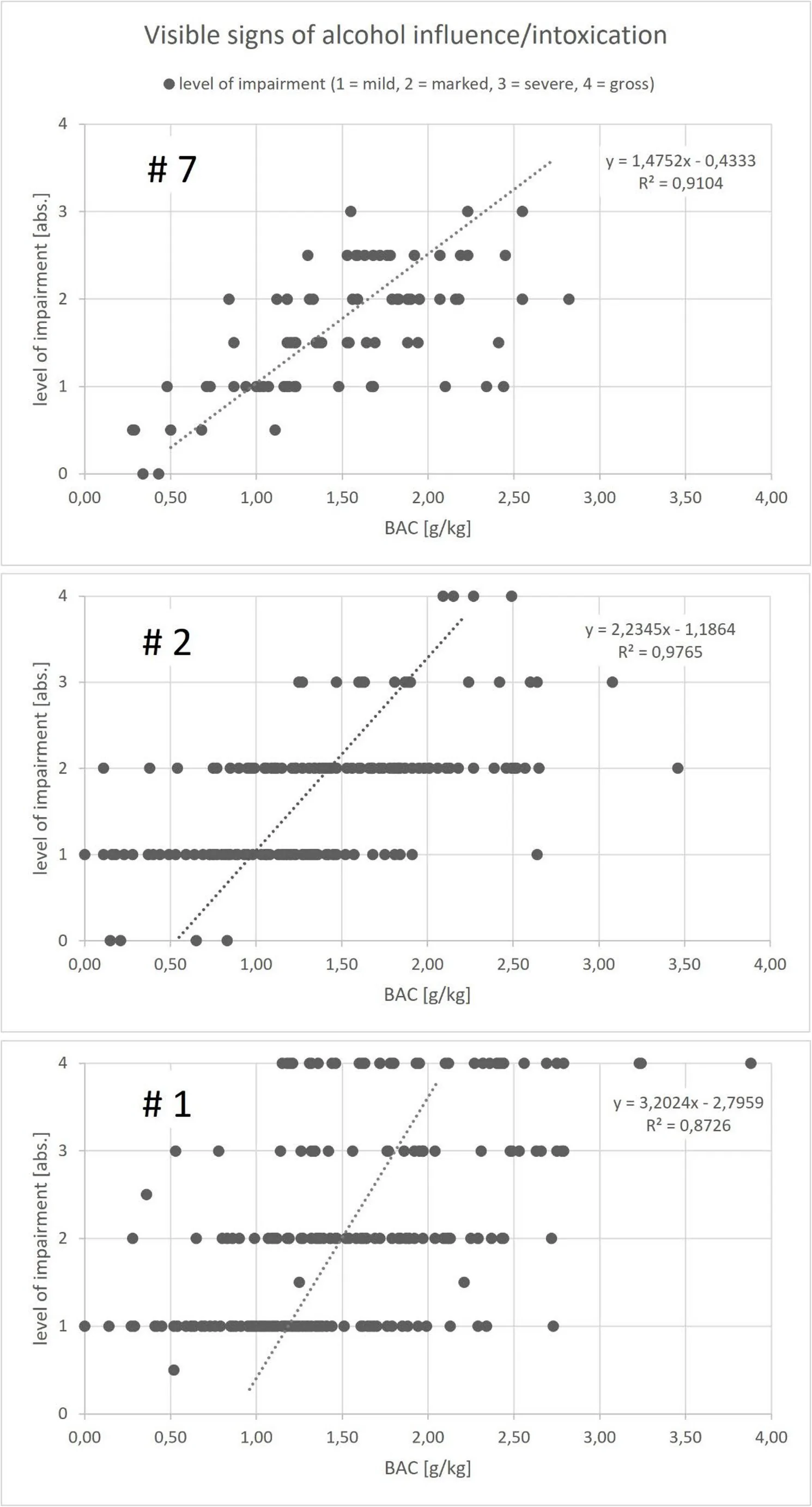
Correlation between global assessment of impairment and forensic BAC across individual physicians. Examples for good correlation are shown. A slope close to the angle bisector signifies maximal discriminative power (top), a steeper slope (bottom) is synonymous with lower differentiation potential

Conversely, when physicians utilized the full range of impairment levels—especially if further supplemented by intermediate grades or explicit zero-points—a high degree of correlation was observed (Fig. 6).

Overall, 6 of the 14 contract practitioners exhibited strong correlations with coefficients (R^2^) exceeding 0.9. Two additional physicians demonstrated a weak correlation that allowed for the determination of R^2^, although these values remained below 0.9. In the remaining six cases, the determination of a correlation coefficient was not possible; for four of these practitioners, no correlation whatsoever could be established between the measured BAC and their clinical medical evaluation.

Table 2 summarizes the correlation equations and coefficients for individual contract physicians, illustrating the variable degree of concordance between clinical assessments and forensic BAC.

**Table 2:** Correlation equations and coefficients of contract physicians

Physician	Correlation equation	Correlation coefficient (*R*_2_)
*# 1*	y = 3,2024x − 2,7959	0,8726
*# 2*	y = 2,2345x − 1,1864	0,9765
*# 3*	y = 1,372x − 0,5633	-
*# 4*	-	-
*# 5*	y = 1,3114x − 0,36	-
*# 6*	y = 1,3667x + 0,3184	0,9656
*# 7*	y = 1,4752x − 0,4333	0,9104
*# 8*	y = 3,6755x − 3,4994	0,9428
*# 9*	-	-
*#10*	y = 1,0553x + 0,376	0,9431
*#11*	y = 1,6182x − 0,5018	0,9205
*#12*	-	-
*#13*	-	-
*#14*	y = 4,3079x − 3,7573	0,6979

### Case study: physician # 4 – systematic diagnostic fixation

**Fig. 7:**
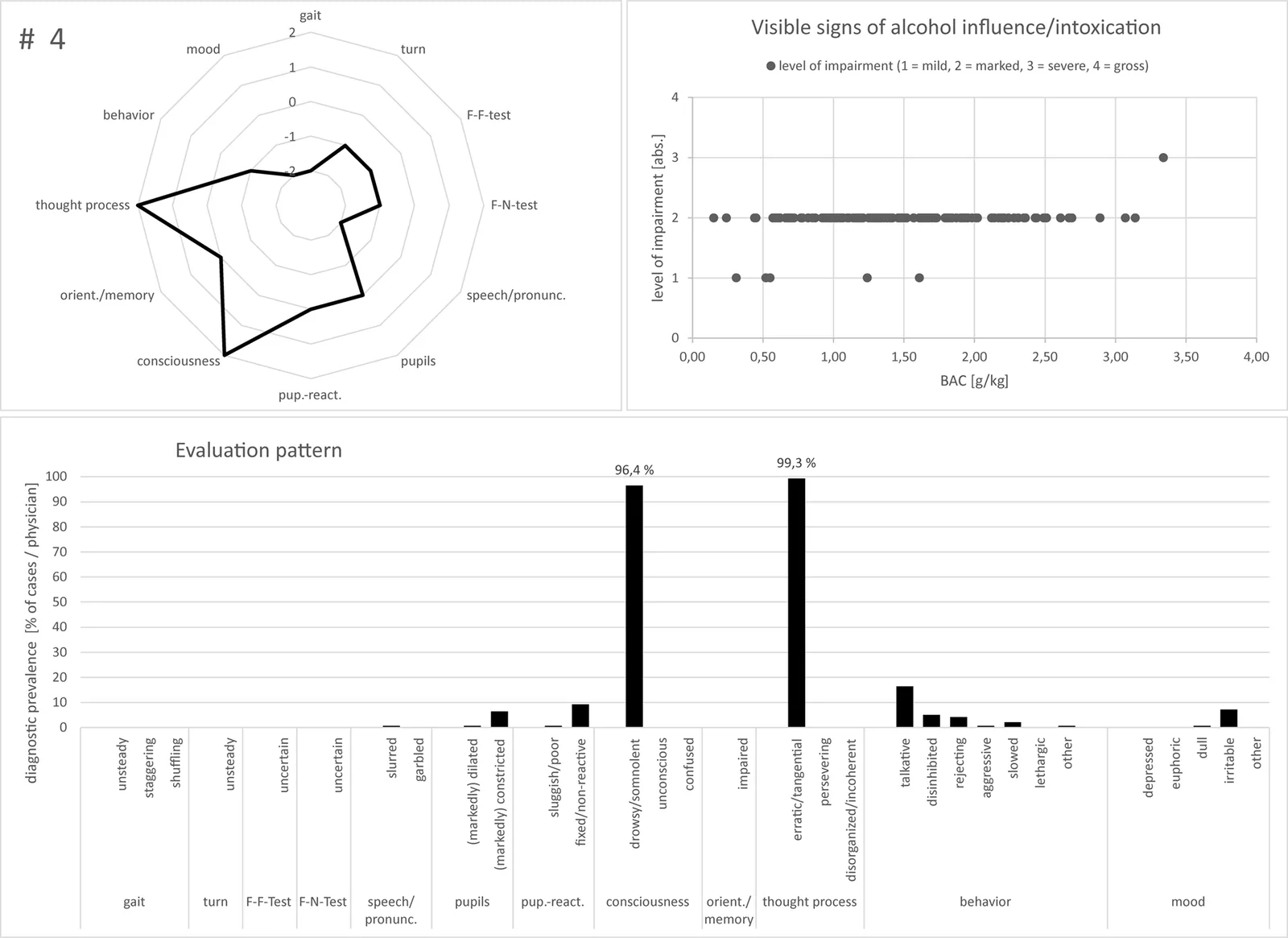
Example for a highly anomalous diagnostic pattern. A combination of extreme over- and underreporting results in a massive deformation of the radar plot polygon, demonstrating negligible concordance with peer group standards (*top left*). Furthermore, a near-total restriction to a single impairment grade precludes any correlation between the clinical assessment and the analytical BAC (*top right*). The bar plot (*bottom*) is indicative of a highly schematic evaluation pattern. For clarity, this plot depicts only the percentage of documented abnormalities for each parameter, while the corresponding frequency of unremarkable findings was omitted to maintain legibility

A detailed analysis was conducted on a prominent negative outlier identified in the heatmap (Table 2). This practitioner presented with the highest cumulative underreporting score (-10) combined with significant overreporting (+ 4). Due to this unique constellation of biases, the data polygon in the physician’s radar plot is severely skewed, indicating negligible overlap with the peer group’s assessment standards. Furthermore, a near-total restriction of evaluations to a single impairment level resulted in a complete absence of correlation between the medical assessment and measured BAC. Further investigation revealed a highly schematic approach: nearly 100% of this physician’s cases were diagnosed with “drowsy/somnolent” consciousness and “erratic/tangential” thought processes (Fig. 7).

### Analysis of test omission rate

**Fig. 8:**
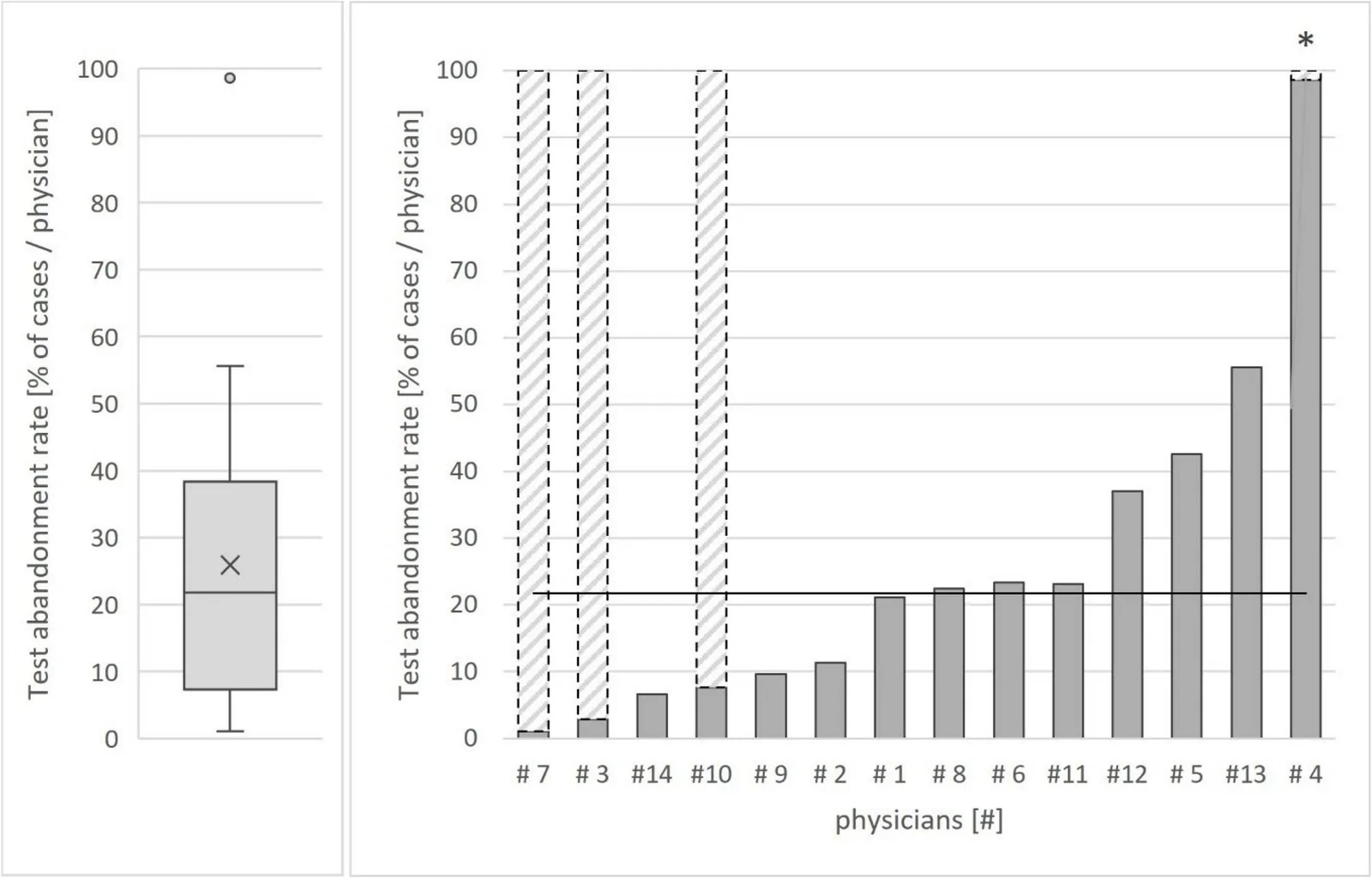
Documented (*dark*) and inferred (*hatched*) test omission rates among experienced contract physicians. *Solid bars* represent documented omission rates (dTOR), while *hatched* segments indicate inferred omissions (iTOR). Physician #4 (*) explicitly documented test abandonment (gait, turnaround, finger-finger and finger-nose coordination tests) in nearly all cases without providing justification. In contrast, Physicians #3, #7, and #10 did not disclose test omissions; however, systematic data absence suggests categorical abandonment of one or more specific motor tests (# 3: gait, turn; # 7: turn, F-F-test, F-N-Test, #10: turn). Consequently, the respective iTOR was defined as 100%

This analysis focused on motor skills tests and primarily evaluated the documented test omission rate (dTOR), defined as the explicit recording of test abandonment by a physician. Across the cohort, dTOR values ranged from 1.1% to 98.6%, with a median of 21.8% (Fig. 8, *box plot*). Individual dTOR values are further detailed in Fig. 8 (*bar plot*), with the cohort median indicated by a horizontal line. Notably, Physician #5 abstained from providing a final evaluation of impairment in every instance where a dTOR was documented, representing approx. 43% of this practitioner’s total caseload.

Physician #4 explicitly documented the omission of motor tests in 98.6% of cases without providing justifying circumstances. In the remaining (2 out of 140) cases, the lack of documentation was classified as an oversight, meeting the criteria for “inferred TOR” (iTOR): iTOR was defined as the total absence of recordings for a parameter across a physician’s entire subject cohort and throughout the full observation period. Consequently, the iTOR for Physician #4 was determined to be 100%, visualized as a hatched elongation of the respective bar plot in Fig. 8. Similar evidence of undisclosed test omission was identified in three other physicians (#3: gait and turnaround; #7: turnaround and both finger-finger- and finger-nose tests; #10: turnaround) who otherwise reported low rates of test abandonment. Their iTOR was likewise defined as 100% and depicted as hatched bar extensions (#3, 7 and 10) in Fig. 8.

## Discussion

### BAC distribution

The observed distribution, with median values between 1.33 and 1.78 g/kg, can be explained by the German legal framework: In the context of administrative offenses (i.e., driving a motor vehicle with a blood alcohol concentration of 0.50 g/kg or higher, or a corresponding breath alcohol concentration), a breath alcohol test is sufficient to initiate legal proceedings. In contrast, the threshold for a criminal offense (per se driving impairment under German law) is established at a BAC of 1.10 g/kg or above and mandates confirmation by a blood test; a breath alcohol test alone is insufficient in these, as well as in all other criminal cases. The distribution of blood alcohol concentrations reported herein reflects these circumstances: Blood samples for subsequent alcohol analysis are typically taken when police officers suspect a blood alcohol concentration exceeding 1.10 g/kg, when a suspect refuses breath alcohol testing, or in any other case involving suspected criminal conduct.

### Correlation of impairment level and BAC

Although the relationship between BAC and ordinal impairment ratings combines continuous and categorical data, linear regression is utilized here as a deliberate analytical framework: The medical report form inherently implies a linear relationship between BAC and clinical impairment scores and is designed to communicate a physician’s findings to judicial authorities as a coherent, graded scale. Consequently, we employed linear regression not merely as an estimation of the relationship, but as a diagnostic tool to identify deviations from this intended clinical standard. In contrast, a non-parametric approach like Spearman’s rho would, by design, mask the very inconsistencies (e.g., non-linear usage of the scale or ‘flattening’ of the assessment) that we intend to identify. For the purpose of forensic quality control, the regression model’s ability to measure adherence to a predefined linear standard was deemed more insightful than a mere assessment of monotonicity.

To further strengthen this analytical strategy, we aggregated median BAC values for each impairment level. This approach accounts for biological variability – such as individual alcohol tolerance or extraneous clinical factors – while minimizing the impact of non-linear outliers. This methodology is supported by our large sample size (*N* = 1458) and the Central Limit Theorem. Although this practice reduces available information and may inflate goodness-of-fit, it offers a pragmatic and sufficiently robust framework for detecting intra-observer inconsistencies: It effectively identifies instances where clinicians fail to capture the linear progression of impairment and thereby facilitates meaningful inter-observer comparisons.

Within this model, the ideal medical assessment should exhibit a correlation slope approximating 1.0 – closely following the angle bisector – for maximum discriminative power, complemented by a high R^2^. However, only six of the 14 evaluated physicians approached this ideal. A comprehensive utilization of the entire available grading scale, ideally including intermediate steps and zero-points, appears to be a primary prerequisite and predictor for a robust correlation between analytical data and clinical evaluation. Conversely, several practitioners exhibited a markedly restricted assessment range, often limited to only one or two (of five available) impairment levels. This led to a complete lack of correlation in four out of 14 panel physicians. Smaller biases resulted in intermediate correlations for the remaining four practitioners, only two of which provided enough data points to allow for the calculation of R^2^. These findings align with previous research indicating similarly weak correlations between BAC and clinical assessments in a traffic setting [14, 15].

### Bias in the reporting of individual criteria

While five physicians (#1, 10, 12, 13, 14) showed no relevant cumulative bias, the overall median was skewed by the significant deviations of their colleagues (see below). Consequently, an objectively accurate finding may appear as a slight trend (± 1) simply due to the group’s shifted average. Conversely, slight tendencies toward over- or underreporting may likewise be masked.

Significant bias was evident in nine practitioners, with physician #4 representing a “worst-case scenario” due to a combination of severe under- and overreporting. Among the eight others with significant bias, physicians #3, 5, and 7 demonstrated systematic underreporting, while physicians #2, 6, 8, 9, and 11 showed systematic overreporting.

Such bias is particularly critical when it affects “hard” criteria essential for the legal assessment of driving unfitness or criminal responsibility, such as gait, speech, consciousness, and thought processes (e.g., Physicians #4 and #2). Notably, physician #3 documented abnormalities in speech and motor skills less frequently than the peer group, despite examining a cohort with a statistically higher median BAC. This suggests a more profound underreporting of core forensic parameters than is immediately apparent from the radar plots alone.

### Test omission rates

While this study primarily evaluated documented test abandonment (dTOR), the detection of absolute underreporting in four physicians necessitated the definition of “inferred TOR” (iTOR). It should be noted that actual omission rates likely exceed the estimates, as sporadic and undisclosed test abandonment, where a parameter was left blank without being explicitly marked as ‘not performed’, remained undetectable within the used methodology. Although instructional variations – such as highlighting the right to refuse – undoubtedly impact participation, this factor alone is insufficient to explain the categorical omission of specific tests observed in some physicians. Instead, financial incentives (such as time-saving measures in a per-case reimbursement system) must also be considered. Ultimately, clinical evaluations that fail to disclose omitted tests lack forensic integrity and may have distorted the data presented here by masking systematic overreporting.

### Implications for forensic routine

The extent of the identified medical bias is highly problematic, as clinical reports are rarely scrutinized and generally carry more evidentiary weight than police observations. Extreme over- or underreporting in high-impact parameters is especially critical: For example, a physician who habitually documents impaired consciousness and erratic thought processes effectively establishes a compelling basis for criminal liability (e.g., unfitness to drive – in Germany a punishable crime under Sect. 316 StGB, the German criminal code) or, conversely, provides grounds for diminished culpability (according to Sect. 20/21 StGB) for virtually every single subject.

Beyond such extremes, the majority of routinely employed contract physicians exhibit assessment patterns that pose significant challenges for judicial processing. Limited usability of medical reports appears to be the rule rather than the exception in forensic-toxicological casework. Based on our data, only approximately 50% of cases (handled by physicians #1, 6, 7, 8, 10, 11, 14) are evaluable without significant limitations due to bias, TOR, or poor correlation. Approximately 40% are of limited use, and nearly 10% (Physician #4) are entirely unusable for forensic expert opinions. These figures are particularly concerning given that they represent experienced contract physicians from whom a high degree of validity is expected. The findings are especially striking as the evaluation was limited to BAC, where a well-established dose-response correlation should result in high clinical concordance. Consequently, this status quo compromises constitutional principles of equal treatment and severely restricts research into safety thresholds (e.g [16]). Furthermore, inconsistent medical evaluations counteract efforts to unify standardized field sobriety testing by law enforcement. While rigorous quality management systems ensure high-precision laboratory analytics, pre-analytical deficiencies in clinical assessments cannot be corrected post-hoc.

### Recommendations and future research

To address the uncovered deficiencies, a shift from “formulaic” reporting toward individualized clinical assessment utilizing the full test battery and grading spectrum is required. Standardized forms should be adjusted to mandate the explicit documentation of test completion or the specific reasons for omission (e.g., non-compliance).

Until these improvements are realized, the presented results provide a compelling argument for the use of police bodycams to record subject behavior [17], allowing for later objective review by forensic-toxicological and/or traffic medical experts. At the very least, if there are specific indications that a traffic-related criminal offense has been committed, the use of bodycams should be considered.

Future research should quantify inter-observer variability using standardized video material and explore the impact of “prompting” – specifically, if and how a physician’s assessment changes when provided with an alleged (high or low) preliminary breath alcohol or drug test result.

Finally, to align clinical practice with the rigorous quality control measures mandated for forensic laboratories, a specialized qualification program for physicians performing forensic blood draws is essential. This should include formal certification, recurrent training in standardized assessment protocols, and proficiency testing to ensure competencies in both clinical examination techniques and forensic documentation. Ultimately, standardized assessment protocols – uniformly structured and mandatory – are required to ensure the validity of forensic evaluations.

### Limitations

Several limitations must be considered when interpreting the results of this study. Most fundamentally, while the subject cohorts to each physician were largely comparable regarding median BAC, they were not identical, which may introduce minor demographic or physiological variances across the collectives. In this context, instances of apparent overreporting may be partially attributable to the undetected influence of other psychoactive substances, as the forensic analysis was restricted solely to BAC.

Methodologically, the reliance on linear regression for ordinal data warrants careful interpretation. While the central limit theorem supports the robustness of parametric models in a large sample (*N* = 1458), the inherent subjectivity of clinical assessments remains a relevant factor. Additionally, calculation of regression coefficients and R² values based on median BAC values aggregated within impairment categories inevitably reduces information and may influence goodness-of-fit. However, while employing non-parametric methods – such as ordinal regression – might offer valuable insights, these approaches would likely obscure the specific intra-observer inconsistencies we aimed to detect. For the purpose of forensic quality control, the linear model was therefore considered the more insightful diagnostic tool, despite the inherent trade-offs.

Furthermore, a foundational decision was made to evaluate reporting habits based on the frequency of negative rather than positive findings. This approach was necessitated by the inconsistent availability of multiple-choice options across different parameters, which precluded a uniform comparative analysis of positive indicators. It is important to note, however, that “negative findings” in this context inherently include cases in which tests were simply not performed. Since the frequency of test omissions is not evenly distributed among physicians, the baseline for comparison is inevitably affected.

This issue is exacerbated by the lack of stringent or standardized documentation regarding adherence to the test panel. The absence of such data made it impossible to calculate more precise metrics, such as the rate of negative findings relative exclusively to the number of tests actually performed. Consequently, the data for physicians with a high rate of undeclared test abandonment may be skewed toward a profile of underreporting. Conversely, test omissions may also mask an underlying trend toward overreporting that would otherwise be apparent. To address these uncertainties, further research is required to elucidate inter-observer variation, ideally through the comparative evaluation of standardized video material – with and without experimental prompting – to better identify the mechanisms of bias.

## Data Availability

Due to the sensitive nature of the information, the raw data supporting the findings of this study are not publicly available. Data are stored on a controlled storage device at the Institute of Legal Medicine in Essen and may be made available by the corresponding author upon reasonable request.
